# [Corrigendum] Calcium efflux from the endoplasmic reticulum regulates cisplatin‑induced apoptosis in human cervical cancer HeLa cells

**DOI:** 10.3892/ol.2025.15020

**Published:** 2025-04-07

**Authors:** Luyan Shen, Naiyan Wen, Meihui Xia, Yu Zhang, Weimin Liu, Ye Xu, Liankun Sun

Oncol Lett 11: 2411–2419, 2016; DOI: 10.3892/ol.2016.4278

Subsequently to the publication of the above paper, an interested reader drew to the Editor's attention that, for the confocal microscopy images shown in Fig. 2A on p. 2414, the data shown in the top row (the ‘12 h’ data) for the ‘Cisplatin + BAPTA/A’ and ‘Cisplatin + 2-APB’ experiments were strikingly similar.

The authors consulted their original data, and realized that this figure had inadvertently been assembled incorrectly. The revised version of Fig. 2, now showing the correct data panel for the ‘Cisplatin + 2-APB’ experiment, is shown on the next page. The authors regret the error that was made during the preparation of this figure, although they were able to confirm that this error did not seriously affect the conclusions reported in the paper. The authors are grateful to the editor of *Oncology Letters* for allowing them the opportunity to publish this Corrigendum; furthermore, they apologize to the readership for any inconvenience caused.

## Figures and Tables

**Figure 2.**
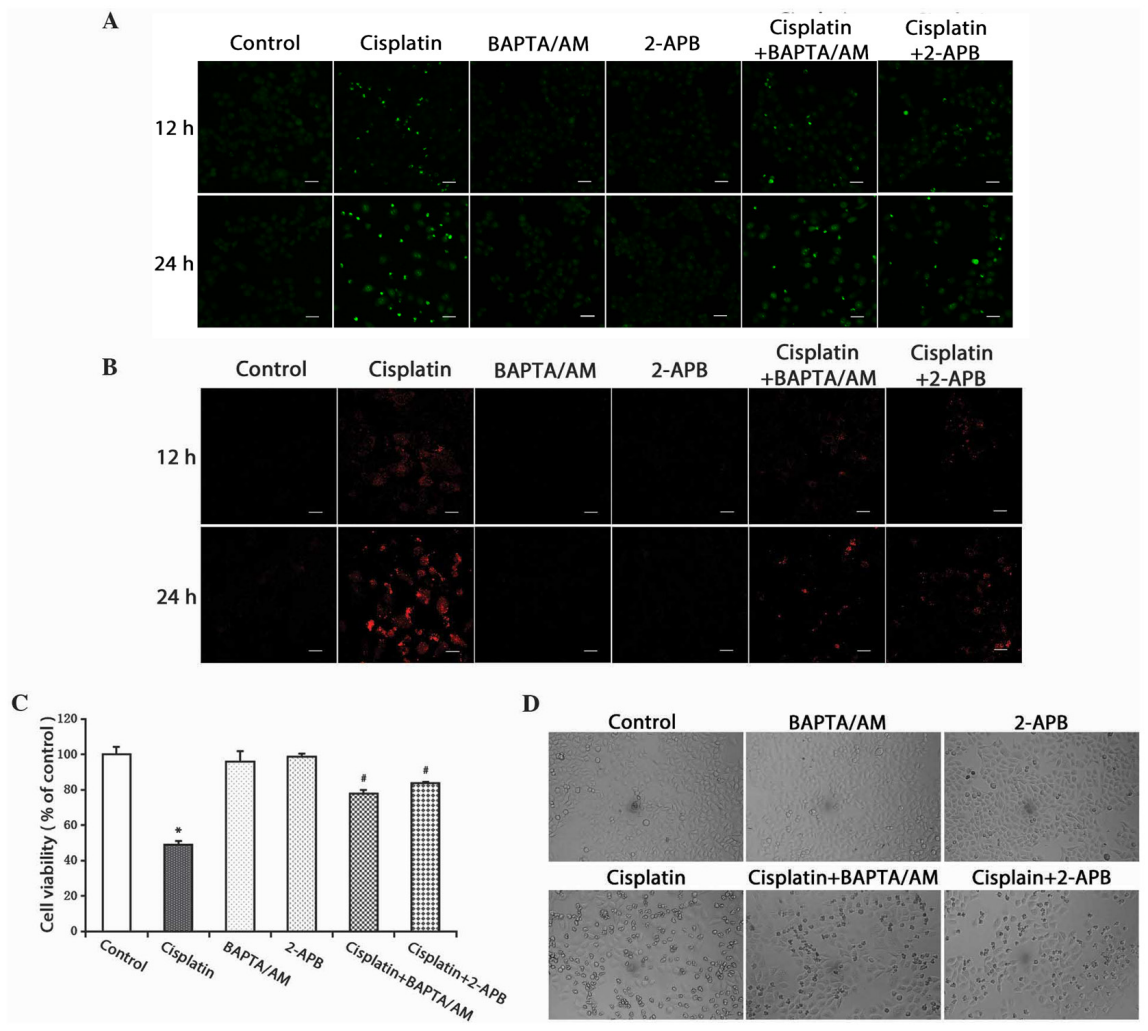
Inhibition of calcium signaling decreases the level of free Ca^2+^ in the cytosol and mitochondria, and inhibits cell growth. (A) HeLa cells were treated with cisplatin (5 µg/ml) with or without BAPTA/AM (2.5 µM) and 2-APB (100 µM) for 12 and 24 h. The cells were incubated with the fluorescent calcium indicator, Fluo-4/AM. Calcium concentrations in the cytosol were observed by confocal microscopy (scale bar, 40 µm). (B) HeLa cells were treated with cisplatin (5 µg/ml) with or without BAPTA/AM (2.5 µM) and 2-APB (100 µM) for 12 and 24 h, and incubated with the fluorescent calcium indicator, Rhod-2. Calcium concentrations in the mitochondria were observed by confocal microscopy (scale bar, 30 µm). (C) HeLa cells were treated with cisplatin (5 µg/ml) with or without BAPTA/AM (2.5 µM) and 2-APB (100 µM) for 24 h. Cell viability was determined using the 3-(4,5-dimetrylthiazol-2-yl)-2,5-diphenyltetrazolium bromide assay. Data are presented as the mean ± standard deviation (n=3). *P<0.05 vs. control; ^#^P<0.05 vs. cisplatin. (D) HeLa cells were treated with cisplatin (5 µg/ml) with or without BAPTA/AM (2.5 µM) and 2-APB (100 µM) for 24 h. Cell morphology was observed using an inverted phase contrast microscope at ×100 magnification. BAPTA/AM, bis-(o-aminophenoxy)ethane-N,N,N’,N’-tetra-acetic acid acetoxymethyl ester; 2-APB, 2-aminoethyl diphenylborinate.

